# Protective Effect of Aromatic Plant Essential Oil Administration on Brain Tissue of PTZ-Treated and Non-Treated Mice

**DOI:** 10.3390/ijms26199618

**Published:** 2025-10-02

**Authors:** Olga Pagonopoulou, Eleni Koutroumanidou, Achilleas Mitrakas, Aglaia Pappa, Georgia-Persephoni Voulgaridou, Despoina Vasiloudi, Sofia-Panagiota Alexopoulou, Triantafyllos Alexiadis, Maria Lambropoulou

**Affiliations:** 1 Laboratory of Neurophysiology, Medical School, Democritus University of Thrace, 68100 Alexandroupolis, Greece; elenkoutrou84@gmail.com; 2 Laboratory of Histology–Embryology, Medical School, Democritus University of Thrace, 68100 Alexandroupolis, Greece; amitrak@med.duth.gr (A.M.); talexiad@med.duth.gr (T.A.); mlambro@med.duth.gr (M.L.); 3 Department of Molecular Biology and Genetics, Democritus University of Thrace, 68100 Alexandroupolis, Greece; apappa@mbg.duth.gr; 4 Laboratory of Physiology, Medical School, Democritus University of Thrace, 68100 Alexandroupolis, Greece; gvoulgar@med.duth.gr (G.-P.V.);

**Keywords:** epilepsy, PTZ, essential oils, *Menta piperita*, antioxidant, ROS, *Mentha pulegium*, *Mentha spicata* wild, *Mentha piperita*, *Lavandula angustifolia*, *Origanum dictamnus*, DPPH, MDA

## Abstract

Epilepsy manifests as recurrent spontaneous seizures associated with irregular brain activity. Recognizing the limitations of conventional antiepileptic treatments, we explored the therapeutic potential of essential oils (EOs) derived from Greek aromatic plants (*Mentha pulegium*, *Mentha spicata* wild, *Mentha piperita*, *Lavandula angustifolia* and *Origanum Dictamnus*). Specifically, we explored their radical scavenging capacity (DPPH), as well as their antioxidant (AOP and MDA levels) and neuroprotective effect in a PTZ-induced epilepsy Balb/c mice model (animals were pretreated with EOs prior to PTZ treatment). Our results indicated that *Mentha piperita* emerges as the most promising EO, demonstrating strong antioxidant activity and the highest radical scavenging ability (IC_50_ = 1.9 mg/mL). *Mentha pulegium* also exhibited considerable antioxidant potential, demonstrating the strongest effect in the AOP assay when administered prior to PTZ treatment. Furthermore, *Origanum dictamnus* exhibited the strongest potential to attenuate MDA formation in the presence of PTZ. Finally, immunohistochemistry indicated a trend of neuronal preservation in animals pretreated with EOs prior to PTZ, with *Mentha piperita* demonstrating the most significant effect. Based on these findings, we suggest that certain EOs possess significant antioxidant and neuroprotective properties. Further research is warranted to validate these results and elucidate the active ingredients responsible for the observed properties.

## 1. Introduction

Oxidative stress refers to a phenomenon characterized by an imbalance between the generation of reactive oxygen species (ROS) and the ability of the cellular antioxidant mechanisms to neutralize them. ROS attack a variety of cellular components, including DNA and proteins, causing oxidative damage that may contribute to cellular dysfunction and death. Oxidative damage is being recognized as a major factor in the development of neurotoxicity and in the pathophysiology of neurodegenerative disorders, including epilepsy [1,2].

Previous studies have reported a correlation between ROS overproduction and aggregation of misfolded proteins. For example, the amyloid-beta, a hallmark protein in patients with Alzheimer’s disease, has been associated with the induction of ROS production, while the mitochondrial dysfunction, which is observed in Parkinson’s disease, is linked to ROS-induced oxidative damage. In addition, damaged neurons further contribute to increased ROS generation, leading to a feedback loop that could worsen the medical status of the patient. The role of ROS as signalling inflammatory molecules constitutes another linkage, highlighting the multifaceted interplay among ROS and neurodegenerative diseases as well as in epileptic syndromes [3]. Epilepsy refers to a group of clinical features characterized by recurrent and spontaneous seizures. The estimated prevalence of epilepsy is near 1–2% of the global population [4]. A variety of pathophysiological mechanisms are related to the development of epilepsy and include distinct neurotransmitter systems like GABAergic and glutamatergic pathways. Furthermore, oxidative stress plays a crucial role in further enhancing the neuronal toxicity associated with the disease. Specifically, high levels of ROS have been shown to result in cellular damage, exacerbating the progression of seizures. Numerous anticonvulsant medications are available; however, over 20% of cases are resistant to current treatments, while the synthetic origin of drugs is related to severe side effects. The in-depth understanding of the mechanisms underlying oxidative stress and neurodegeneration linkage is of crucial importance for the development of novel therapeutic strategies, considering that current approaches mainly focus on symptom suppression rather than addressing the causative factors [5].

A variety of physical compounds have gained attention for their potential role as antioxidant agents, while their limited side effects emerge as an important advantage. A major category of natural antioxidants is that of essential oils (EOs), extracted from medicinal plants. Specific phytochemical compounds isolated from plant extracts, such as terpenes and flavonoids, exhibit antioxidant activity, free radical scavenging ability and the capacity to neutralize ROS. Consequently, EOs can be used as factors against ROS overproduction and thus, the generation of oxidative stress. Moreover, EOs possess well-established anti-inflammatory and neuroprotective properties, while their role in protecting normal mitochondrial function has also been extensively studied [6,7,8].

*Μentha pulegium*, *Mentha spicata* and *Mentha piperita* are three members of *Mentha* genus, known as pennyroyal, spearmint and peppermint, respectively. They possess unique characteristics, such as anti-cancer, anti-obesity and anti-microbial properties [9,10,11]. For instance, certain studies demonstrated the anti-inflammatory activity of *Μentha pulegium* [12], while *Mentha spicata* has been attributed with a wide range of bioactive effects, including antidiabetic, diuretic antioxidant and antiparasitic [13]. Along these lines, *Mentha piperita* has been attributed with wound-healing, cardioprotective and antiviral activities [14]. *Origanum dictamnus* (also known as Cretan or Greek oregano) contains two very potent phytochemicals, p-Cymene and Carvacrol [15], while it exhibits antiaging and antiproliferating effects [16,17]. Finally, *Lavandula angustifolia*, known as English lavender, was also included in our research, due to its content of bioactive compounds, such as Eucalyptol, Linalool and Camphor, and its anti-inflammatory properties as well as its putative neuroprotective effects [18,19]. All of the used EOs are derived from aromatic plants, which thrive in Greece, are used in aromatherapy and are a part of the Mediterranean diet [15].

It should be noted that while previous studies have reported the antioxidant activity of these plants, this property has not been extensively studied, especially in the context of neurotoxicity [13,16,17]. Therefore, the scope of this study was to explore the role of EOs derived from these five native aromatic plants in the management or treatment of epilepsy by assessing their ability to act as radical scavenging factors, to mitigate oxidative stress and to protect against neurotoxicity in a PTZ-induced epileptic model.

## 2. Results

### 2.1. Evaluation of the Cell-Free Radical Scavenging Capacity Through DPPH Assay

**Figure 1:**
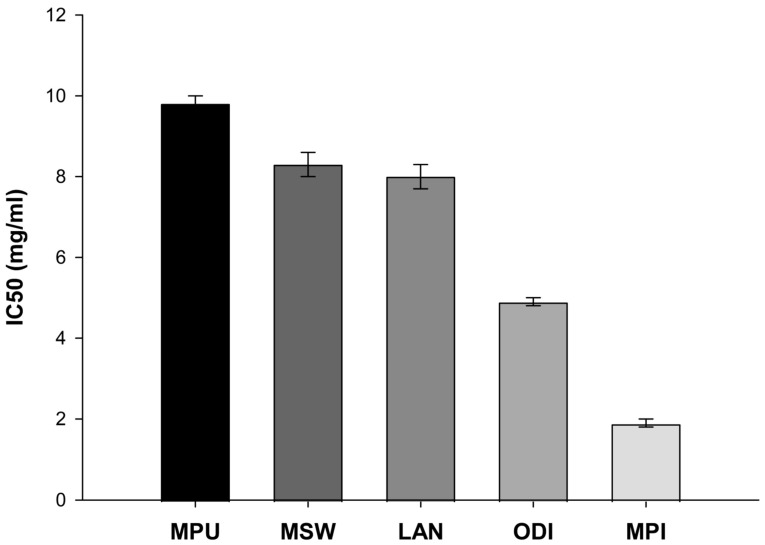
Radical scavenging activity of the EOs through a DPPH assay. MPU: *Mentha pulegium*, MSW: *Mentha spicata* wild, LAN: *Lavandula angustifolia*, ODI: *Origanum dictamnus*, MPI: *Mentha piperita*. Data are representative of IC_50_ mean values ± SD of three independent experiments for each condition tested.

The ability of the studied EOs to inhibit the generation of free radicals was evaluated through the DPPH assay. With this assay, the IC_50_, thus the EOs concentration at which 50% of the initial DPPH free radical is effectively scavenged, was evaluated. Our study showed that *Mentha piperita* exhibited the lowest IC_50_ value (1.9 mg/mL) and thus, the strongest free radical scavenging effect among the examined EOs (Figure 1). Moreover, *Origanum dictamnus* appeared to exert moderate antioxidant capacity with an IC_50_ value of 4.9 mg/mL. Finally, the IC_50_ values of *Lavandula angustifolia* (8.0 mg/mL), *Mentha spicata* wild (8.3 mg/mL) and *Mentha pulegium* (9.8 mg/mL) were relatively high and thus, exhibited limited radical scavenging potential.

### 2.2. Antioxidant Power Study

The antioxidant potential of each EO was determined using a uric acid standard curve. The antioxidant potential was determined in the control group, the PTZ-treated group, the EOs-treated groups and in groups pre-treated with EOs and then further treated with PTZ. A higher value of uric acid equivalent represents stronger antioxidant protection capacity.

**Figure 2:**
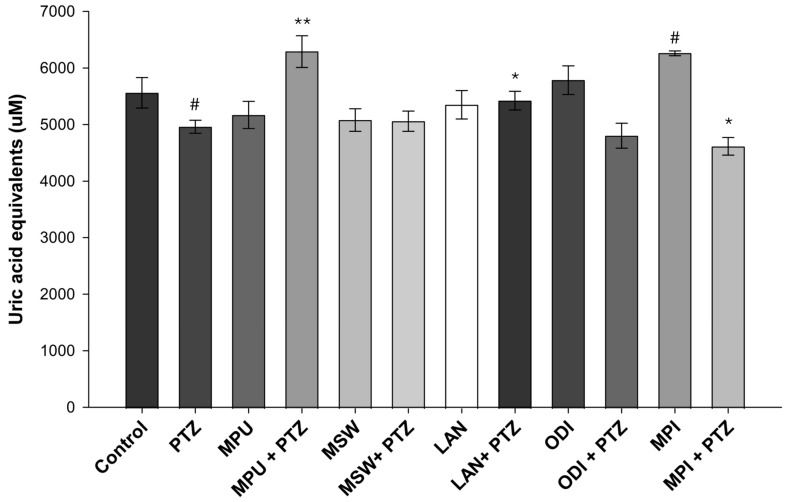
Total antioxidant potential of EOs without and with PTZ treatment. Data are representative of IC_50_ mean values ± SD of three independent experiments for each condition tested (MPU: *Mentha pulegium*, MSW: *Mentha spicata* wild, LAN: *Lavandula angustifolia*, ODI: *Origanum dictamnus*, MPI: *Mentha piperita*). Note: # *p* ≤ 0.05, vs. control,* *p* ≤ 0.05, ** *p* ≤ 0.01 vs. PTZ.

Our data demonstrated that PTZ significantly reduced the antioxidant capacity of the samples in a statistically significant manner (*p* = 0.0241). Interestingly, *Mentha pulegium* exhibited lower antioxidant potential than the control (not statistically significant; however, *p* = 0.1345); nevertheless, its antioxidant role appeared to be significant after PTZ administration (*p* = 0.0016). A slight increase in the antioxidant potential was observed in groups pre-treated with *Mentha spicata* wild (*p* = 0.4713) and *Lavandula angustifolia* (*p* = 0.0167) prior to PTZ administration. *Mentha piperita* demonstrated a statistically significant, strong increase in the antioxidant capacity of the sample when administered alone (*p* = 0.0114); however, in the samples where it was combined with PTZ administration, the antioxidant potential was significantly reduced (*p* = 0.0361). A similar pattern, not statistically significant, however, was observed with the *Origanum dictamnus* EO (*p* = 0.3562 and *p* = 0.3312 when used alone or with PTZ, respectively) (Figure 2).

### 2.3. Evaluation of Lipid Peroxidation via Malondialdehyde (MDA) Levels

**Figure 3:**
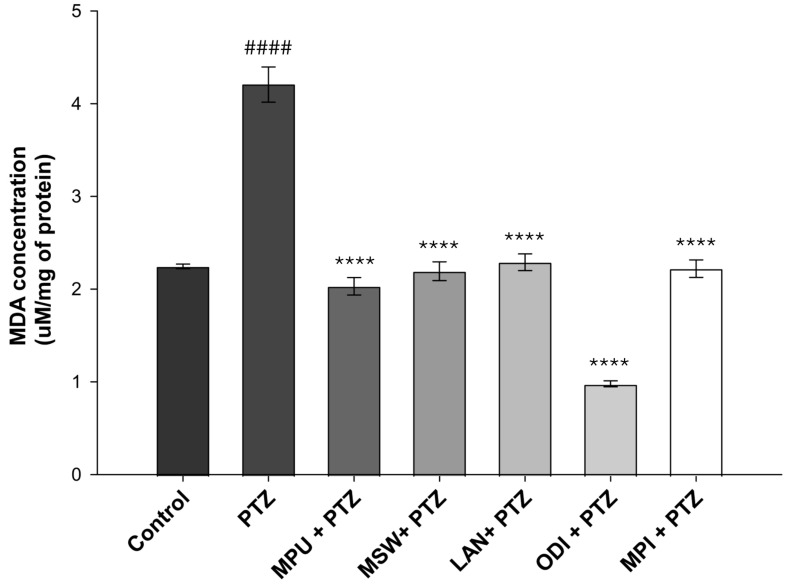
Lipid peroxidation levels as indicated by the concentration of MDA in control, PTZ-treated and EO-pretreated/PTZ-treated samples. Data are representative of IC_50_ mean values ± SD of three independent experiments for each condition tested (MPU: *Mentha pulegium*, MSW: *Mentha spicata* wild, LAN: *Lavandula angustifolia*, ODI: *Origanum dictamnus*, MPI: *Mentha piperita*). Note: #### *p* < 0.0001 vs. control, **** *p* < 0.0001 vs. PTZ.

The protective potential of EOs was further examined by determining the concentration of MDA, an aldehyde established as a marker of lipid peroxidation. Our data indicated that PTZ administration led to high levels of lipid peroxidation, with the MDA concentrations being doubled in comparison to those of the control group (*p* < 0.0001). All of the examined EOs exhibited statistically significant protective potential, as they managed to greatly decrease lipid peroxidation levels near those of the control group (*p* < 0.0001). Specifically, the EO of *Origanum dictamnus* exhibited the highest protective effect, managing to decrease MDA at approximately half of the control levels, while *Mentha pulegium*, *Mentha spicata*, *Mentha piperita* and *Lavandula angustifolia* also demonstrated significant protective capacity compared to the PTZ group, as they restored lipid peroxidation near control levels (Figure 3).

### 2.4. Immunohistochemical Study

**Figure 4:**
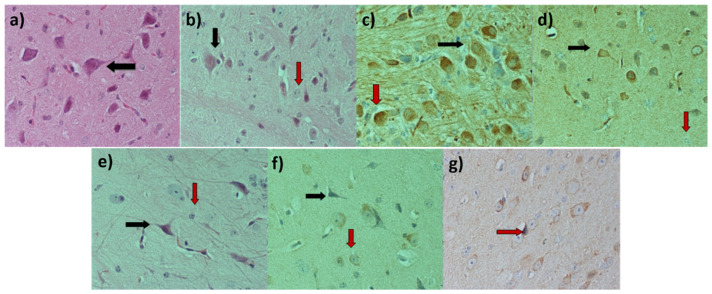
Immunohistochemically treated sections of mouse brains in order to study their nerve cell and glial cell composition after PTZ and PTZ-EOs treatment. In the above image, different treatment groups are presented as follows: (**a**) Control, (**b**) PTZ group, (**c**) *Μentha piperitα* EO + PTZ group, (**d**) *Μentha spicata* wild EO + PTZ group, (**e**) *Origanum dictamnus* EO + PTZ group, (**f**) *Μentha pulegium* EO + PTZ group and (**g**) *Lavandula angustifolia* EO + PTZ group. Black arrows indicate the neurons and red arrows the microglial cells.

Neuron density was evaluated manually at a ×40 magnification per sample, under a Nikon Eclipse 50i light microscope. Positive neurons were counted in at least 10 high-power optical fields at a ×40 magnification, and their total number was divided by the number of optical fields in order to obtain the final score. Figure 4 shows the outcome of this observation, with varying numbers of neurons per field depending on the treatment (as indicated by the black arrows), along with an increase in the number of glial cells in all groups compared to the control. Microglial cells (marked with red arrows) belong to the immune system of the central nervous system and are immediately activated as macrophages in various conditions, such as in peripheral nerve injury, spinal cord injury, inflammation, chemotherapy, etc. [20].

Our results showed that in the samples from PTZ-only-treated animals, there was a marked reduction in the number of neurons per field in comparison to the control group. On the contrary, in all brain samples derived from animals pre-treated with one of the examined EOs, there was a relative protective effect of the EO against the PTZ-induced neuron damage, reflecting the preservation of the viability of additional neurons. Furthermore, the number of glial cells, which act as scavengers and anti-inflammatory agents, increased in both the PTZ- as well as EOs + PTZ-treated groups. However, the small sample size did not allow us to draw statistically significant conclusions; therefore, numbers are not included in this manuscript.

## 3. Discussion

Our main goal in the current study was to evaluate the antioxidant activity of EOs extracted from five aromatic indigenous plants from Greek flora. To achieve this, a DPPH assay was performed, where the EO of *Menta piperita* exhibited the highest ability to scavenge the DPPH radical. Subsequently, we investigated the antioxidant and neuroprotective effects of EO administration in the brains of PTZ-treated Balb-c mice. PTZ is a compound known for its ability to induce oxidative stress through enhancing lipid peroxidation, thus increasing MDA levels and attacking antioxidant enzymes such as glutathione and superoxide dismutase [21,22,23]. PTZ-induced seizures are directly related to the increased production of the amino acid GABA, the following GABAergic neuronal damage and, ultimately, the death of neurons. Furthermore, PTZ acts as an antagonist of GABA-A receptor and induces severe and acute epileptic seizures as well as high levels of inflammation and neurotoxicity when administered to Balb/c mice. Consequently, it is widely used as a common compound for induced animal models of epilepsy [24,25,26]. The evaluation of total antioxidant capacity highlighted the EO of *Mentha piperita* as the most potent when administered alone, while *Mentha pulegium* not only retained but also managed to significantly increase antioxidant power when administered prior to PTZ treatments. Regarding MDA levels, all examined EOs appeared to successfully suppress the PTZ-induced lipid peroxidation increase, evident in the PTZ group. Specifically, *Origanum dictamnus* exhibited the highest efficacy with levels of MDA even lower than those of the control sample. Finally, following our previous study on the suppressive role of specific essential oils in a PTZ animal model of epilepsy [15], we performed an immunohistochemical study with the histological preparations of brain tissues from the animals that had survived the PTZ model. Results revealed a reduced number of neurons per field under the effect of PTZ, as well as an increase in the presence of glial cells. As glial cells constitute a part of the central nervous immune system, their presence in the observation field was anticipated. While almost all oils showed the capacity to inhibit neuronal loss due to PTZ, the small sample size prevented us from drawing statistical conclusions.

The findings of this study may hold clinical perspectives, since they support the idea of incorporating herbal extracts in the management and/or treatment of epilepsy and neurodegenerative diseases. Our previous study, in accordance with this research [15], indicated that *Mentha piperita*, *Origanum dictamnus* and *Mentha pulegium* exert a significant protective role in the PTZ animal model. All three managed to maintain the survival of the animals and/or subsequently increase seizure latency. Specifically, animals treated with *Mentha piperita* exhibited 100% survival and no seizures, while the survival and seizure latency were 100% and 240 ± 15 s for *Origanum dictamnus* and 86% and 119 ± 20 s for *Mentha pulegium*, respectively. These data, in combination with our current findings, illustrate the potential of these three EOs and specifically *Mentha piperita* in neuroprotection and the prevention of seizure occurrence in epilepsy. Furthermore, these studies underscore the potential use of *Menta piperita* EO and its components as natural antioxidants that could probably be used, after thorough examination on a larger experimental scale, in the production of synthetic non-pharmacological formulations.

In the present study, the EO of *Mentha piperita* exhibited the highest antioxidant activity against DPPH radicals (IC_50_ = 1.9 mg/mL) compared to other tested EΟs. Besides demonstrating antioxidant activity against DPPH radicals, the EO of *Mentha piperita* also induced the total antioxidant power of the samples when used alone, while it also managed to attenuate PTZ-induced lipid peroxidation to near control levels. In accordance with our results, Schmidt et al. confirmed the antioxidant activity of *Mentha piperita* against the DPPH and hydroxyl radicals [27]. Additionally, Ramkissoon et al. characterized *Mentha piperita* as a potential source of natural antioxidants capable of reducing oxidative stress levels [28], while Mimica-Dukic et al. demonstrated the high antioxidant activity of *Mentha piperita* against hydroxyl radicals via the Fenton reaction, achieving a 24% reduction. A similar pattern was observed for the EOs of *Metha aquatic* L. and *Mentha longifolia* L.; however, DPPH reduction reached 50% only after *Mentha piperita* treatment [29]. Previously published data indicated the protective role of this EO in γ-irradiated mice, associated with the induction of glutathione peroxidase, glutathione reductase, superoxide dismutase and catalase activity, along with a reduction in the formation of malondialdehyde [30]. Moreover, in another study, EO of *Mentha piperita* exhibited a protective role against chemically induced oxidative stress in the liver and kidney [31].

In our study, while the EO of *Mentha pulegium* did not manage to neutralize the DPPH radical, it exhibited high efficiency in increasing the total antioxidant power of the samples when used prior to PTZ treatment and inhibited the PTZ-induced lipid peroxidation. In contrast, Kamkar et al. reported a lack of significant antioxidant activity in the EO of *Mentha pulegium*, [32]. On the contrary, Messaoudi et al. reported that *Menta pulegium* has significant antioxidant actions; however, they also emphasized the crucial role of its chemical compositions, which appear to depend on the geographical collection area [33,34].

Regarding *Mentha spicata* wild, our findings suggest that its EO did not exhibit a strong capacity to inhibit the DPPH radical or to enhance total antioxidant power. However, it managed to inhibit PTZ-induced lipid peroxidation and maintain relatively low MDA levels. Similarly to *Mentha pulegium*, certain studies have reported that their methanolic extracts exhibit higher antioxidant activity than the aqueous ones. Moreover, previous reports indicated that different types of wild mint demonstrate antioxidant activity associated with the concentration of phenolic compounds. Notably, the phenolic content appears to be influenced by the geographic region in which the specific mint grows [35,36]. Previous studies also demonstrated a reduction in CCl4-induced malondialdehyde levels in human lymphocytes pre-treated with EO of *Mentha pulegium* [37].

In our study, the EO of *Origanum dictamnus* exhibited the second-highest scavenging capacity (IC_50_= 4.9 mg/mL) among the EOs examined. Additionally, it increased the total antioxidant capacity of the samples to levels exceeding those of the control group. In contrast, when the oil was administered prior to PTZ, the same effect was not observed. Nevertheless, the EO strongly attenuated PTZ-induced formation of MDA, reducing its levels to approximately half of the control. In agreement with our findings, Couladis et al. also demonstrated the antioxidant activity of ethanolic extracts of *Origanum dictamnus* [38]. The antioxidant and antimicrobial properties of its methanol extracts were further examined by Gortzi et al. using the Rancimat and malondialdehyde methods, in comparison with standard antioxidants such as butylated hydroxytoluene and a-tocopherol [39]. Extracts with high antioxidant activity were encapsulated in liposomes, and the antioxidant activity was estimated again using differential scanning calorimetry (DSC). All samples demonstrated antioxidant and antimicrobial activity, exceeding that of alpha-tocopherol. Interestingly, the methanolic extract of *Origanum dictamnus* exhibited greater activity than the butylated hydroxytoluene, while its encapsulation in liposomes resulted in higher antioxidant and antimicrobial activity [40,41].

Regarding *Lavandula angustifolia*, our experimental results showed that it exhibits weaker scavenging potential compared to the other examined EOs. Consistently, it did not appear to have a significant effect on the total antioxidant power of the samples tested. On the other hand, *Lavandula angustifolia* EO effectively protected against the PTZ-induced increase in the lipid peroxidation levels. Certain studies have highlighted the antioxidant and scavenging potential of *Lavandula angustifolia* mainly through demonstrating its ability to attenuate lipid peroxidation in the linoleic acid system [42,43]. Blažeković et al. also reported the antioxidant activity of various *Lavandula* species and reported a correlation between their antioxidant activity and their polyphenol content [44]. Finally, Hancianu et al. investigated the antioxidant and anti-apoptotic effects of the *Lavandula angustifolia* EO and found that it significantly reduced MDA levels in Wistar rats that had previously undergone a dementia model under the influence of scopolamine [45].

Regarding our immunohistochemical examination, under the influence of PTZ, a decrease in the number of neurons and an increase in the presence of glial cells were observed. Since glial cells have, among other functions, the role of removing dead neurons, an increase in their presence in the observation field is expected [46]. The results suggest a trend of neuronal preservation in animals pretreated with EOs before PTZ administration compared to those who received only PTZ, with *Mentha piperita* EO being the most effective. This observation supports the notion that EOs can inhibit neuronal damage induced by PTZ, in the concept of suspending further neuronal loss caused by PTZ action. Our findings are in accordance with previously published data highlighting the use of *Mentha piperita* in neurological disorders. Specifically, Hanafy et al. showed that *Mentha piperita* oil was able to increase the latency time to the first convulsion and subsequently reduce the number of convulsions in a PTZ-induced and pilocarpine mouse model [47]. Furthermore, Anjum et al. reported that *Mentha piperita* extracts can prevent the motor dysfunctions in a Parkinson’s disease mouse model through their antioxidant function [48].

One of the limitations of our study is the relatively small number of animals, restricting the interpretation of our immunohistochemical findings. Consequently, to validate our results, especially in neuronal protection, additional studies with larger numbers of animals must be carried out to achieve statistical significance. Future directions of our research also include the examination of specific active compounds identified in the most promising EOs. For instance, menthol and menthone from *Mentha piperita*, pulegone from *Mentha pulegium*, and carvacrol and p-Cymene from *Origanum dictamnus* could be investigated individually or in combination in order to characterize their bioactive properties in the epileptic and neurodegeneration context.

## 4. Materials and Methods

### 4.1. Extraction of EOs

*Mentha pulegium*, *Mentha spicata*, *Mentha piperita* and *Lavandula angustifolia* are endemic in North-Eastern Greece and were collected from the region of Thrace. *Origanum dictamnus* was collected from Crete. EOs of the five plants were isolated and characterized using the experimental procedures, which were described in our previous study [15]. Briefly, samples were air-dried and powdered in an electric blender. Then 200 gr of each sample was hydrodistilled (3 h at 100 °C) in a Clevenger type apparatus and dried over anhydrous sodium sulphate. Finally, they were filtered and stored at −20 °C.

### 4.2. Evaluation of Free Radical Scavenging Activity by DPPH Assay

The free radical scavenging capacity of the EOs was evaluated using the DPPH assay. This assay is based on the interaction between the examined substances and the stable, free radical of DPPH and, specifically, the ability of the antioxidant content of the sample to neutralize this radical. The scavenging of DPPH radical results in the decline of the purple colour of the solution, which is measured spectrophotometrically in the spectrum of the molecule (492 nm) with a Microplate Spectrophotometer (XFLUOR4 Version4.51, Tecan, Mannedorf, Switzerland) [49].

The following procedure was performed for each of the tested EOs separately: DPPH solution (0.01 M) was prepared in ethanol (12.7 mL of 100% ethanol) and 190 μL of this solution was added to 10 μL of EO extract in DMSO in four different concentrations: 0.1 mg/mL, 0.01 mg/mL, 0.001 mg/mL, and 0.0001 mg/mL. The samples were incubated for 30 min in dark conditions. As a blank, 10 μL of EOs with 190 μL of DPPH without incubation was used. Both control and the different concentrations of the samples were performed in triplicate. In total, the assay required 24 wells per EO sample. The absorbance was measured at 492 nm, and the rate of DPPH radical inhibition for each sample was calculated as a % calculation with the following formula:

% DPPH radical scavenging activity = 100 × [(ODblank − ODsample)/ODblank)](1)

Ascorbic acid was used as a positive control for the assay (IC_50_: 28 μg/mL).

The IC_50_ values in the DPPH assay were calculated from the respective curves by regression analysis with a four-parameter logistic curve using Sigma Plot Software (v.10) (Systat, San Jose, CA, USA).

### 4.3. Archived Animal Samples

No new animal experiments were performed for this research. Only archived samples obtained from previously published studies were reused [13], according to the 3Rs principles, particularly the reduction in animal use.

The procedures related to animals were previously described in the study in which the original experiments were conducted [13]. Specifically, all procedures complied with the guidelines and the requirements set by Directive 2010/63/EE and PD 56, and adhered to the 3Rs (replacement, refinement and reduction). No animals were subjected to any pain or discomfort.

In the original study, adult female Balb/c mice (approximately 3 months old), weighing near 25 g, were used [13]. Animals were housed on a 12 h day/12 h night cycle at a temperature between 21 and 23 °C with access to tap water and food ad libitum. Animals were divided into ten groups as described in Table 1.

For the treatments, mice were injected intraperitoneally with EOs (1.6 mL/Kg of body weight) (for the injections oils were diluted 1:4 in PBS, pH 7.4). Subsequently, 60 min after the EO treatment, animals in the PTZ and EOs + PTZ groups received an 80 mg/kg of body weight dose of PTZ via intraperitoneal injection (P6500, Sigma-Aldrich, Steinheim, Germany), a potentially lethal dose for the animals [50,51]. Seizure latency and severity results were described in our previous publication [15]. After 24 h, surviving animals from each group were sacrificed, and their brains were collected.

In control animals, PTZ administration was originally followed by clonic convulsions associated with mouth, face and limb movements, then by tonic seizures for approximately 2 min and they died five minutes later, so their brains were collected immediately after their death.

A part of the tissue was fixed with formalin, embedded in paraffin and stored for future immunohistochemical experiments, while another part was homogenized in PBS and stored in −70 °C to be used for future antioxidant and/or metabolic studies. These are the samples included in the present study.

### 4.4. Antioxidant Power Study

The procedure was performed following the protocol of the assay BIOXYTECH^®^ AOP-450TM (OxisResearch; Portland, OR, USA) as given by the manufacturer. The colourimetric method of uric acid, a small molecule with antioxidant activity, was used. The reduction potential of the sample or standard effectively converts Cu^2+^ to Cu^+^, changing the absorption characteristics of the ion. The reduced form of copper selectively forms a stable 2:1 complex with the chromogenic reagent, exhibiting an absorption maximum at 450 nm; thus, the antioxidant capacity of the sample can be determined through the changes in the intensity of the absorbance. A known concentration of uric acid was used for creating a standard curve, with data being expressed as equivalents of uric acid in μM. Higher values of total antioxidant capacity represent higher protection against oxidation by the examined samples [52,53].

Regarding the preparation procedure, the dilution buffer, the copper solution and the stop solution were left at room temperature for 30 min prior to the experiment. For preparing the standard curve of uric acid, a working solution of 2 mg/mL was prepared in deionized water. Serial dilutions of the working solution were then prepared in deionized water at the following concentrations: 0, 0.125, 0.250, 0.500, 1 and 2 mg/mL. As a blank, 200 μL of buffer solution alone was used, while for the sample, 200 μL of diluted oil (1:10 in buffer solution) was used. A first reference measurement was performed (450 nm), and data were recorded. Then, 50 μL of copper solution was added to each well, and the microplate was left to incubate at room temperature for 3 min. Finally, 50 μL of stop solution was added to each well, and a second measurement was performed at 450 nm. The standard curve for uric acid concentration was generated and used for the determination of each sample’s antioxidant power as uric acid equivalents. All measurements were in technical duplicates for each measurement.

### 4.5. Evaluation of Lipid Peroxidation via Malondialdehyde (MDA) Levels

This assay BIOXYTECH^®^ MDA-586 (OxisResearch; Portland, OR, USA) is based on the reaction of one molecule of malondialdehyde (MDA) or 4-hydroxyalkenal (4-HNE) with two molecules of N-methyl-2-phenylindole—which is a chromogenic reagent—to yield a stable chromophore with a maximum absorbance at 586 nm [54].

For preparing the standard solutions, MDA was diluted in deionized water at a ratio of 1:500 (*v*/*v*) to yield a working concentration of 20 μM. The working solution was then serially diluted in deionized water to achieve the following standard concentrations: 0, 0.5, 1, 2, 3 and 4 μM at a final volume of 200 μL for preparing the standard curve analysis.

Two blank samples (200 μL) were also included in the experiment, specifically a maximum blank sample (the one with the highest protein concentration, as illustrated by a standard BCA method) and a minimum blank sample (the one with the lowest protein concentration).

Then, 650 μL of the 1:3 (*v*/*v*) diluted N-methyl-2-phenylindole reagent was added to 200 μL of the unknown samples and 200 μL of the standard solutions. In the blank samples, 650 μL of a mixture with 75% acetonitrile and 25% acetonitrile was added instead.

All samples (unknown, standard and blanks) were mixed through vortex prior to adding 150 μL of the second reactant in each of them. Then, samples were incubated at 45 °C for 60 min and then centrifuged at 15,000 rpm for 10 min to obtain a clear supernatant. The supernatant was then transferred into cuvettes, and the absorbance was measured at 586 nm with a Biochrom Libra S22 UV/visible spectrophotometer (Biochrom, Cambridge, UK). Finally, the standard curve was constructed based on MDA absorption values and their respective known concentrations from the standard curve.

### 4.6. Immunohistochemistry

Immunohistochemistry was performed with the Vectastain elite ABC kit according to the manufacturer’s instructions [55]. Specifically, brain tissue incisions used for this study were sequentially immersed (for 5 min) in xylol, xylol, 100% ethanol, 100% ethanol, 100% ethanol, 96% ethanol and 96% ethanol. Then, after washing, they were incubated in peroxidase (200 mL H_2_O + 6 mL H_2_O_2_). Samples were washed with PBS for 5 min, placed in a microwave for specific time intervals and washed again with PBS for 5 min. Subsequently, they were incubated for 20 min in blocking solution, and, after this step, a neuron-specific enolase (NSE) antibody was added. After 30 min, samples were washed for 5 min with PBS and incubated with the secondary antibody for 30 min. The incisions were then treated with the Vectastain elite ABS reagent for 5 min and washed again. Finally, drops of diaminobenzidine (DAB+) were added to the solution to produce a brown precipitate. Samples were washed with PBS, a drop of hematoxylin solution (200 mL hematoxylin + 10 mL acetic acid) was added to each, and they were re-washed. Finally, the incisions were sequentially immersed (for 5 min) in 96% ethanol, 96% ethanol, 100% ethanol, 100% ethanol, 100% ethanol, xylol and xylol, covered with entellan glue and left to dry for 8–10 h.

The observation of the incisions was made using the optical microscope Nikon Eclipse 50i, integrated with a digital camera Nikon DIGITAL SIGHT DS-L1 (Nikon Corporation, Tokyo, Japan). The neuro-specific enolase characterizes neurons and neuroendocrine cells APUD; these cells are able to recruit the amine precursor as well as synthesize and secrete polypeptide hormones and biogenic amines. The neuro-specific enolase is found in the cells of the diffuse neuroendocrine system and neuronal tissues, which recruit and decarboxylate amine precursors. It is also associated with neuronal differentiation during auto-repair procedures of the nervous system. Enolase-positive neurons were counted in at least 10 high-power optical fields at a ×40 magnification, and their total number was divided by the number of optical fields to obtain the final score.

### 4.7. Statistical Analysis

At least three independent experiments were performed for each condition tested. The values are expressed as the mean ± standard deviation (SD). No statistical analysis was performed for the DPPH assay since the experiment was conducted in order tο have an estimation on the free radical scavenging activity of each EOs separately and not for comparing samples from different experimental conditions. The Sigmaplot Software (version 10) was used for statistical analysis in all other antioxidant assays, where the statistical significance was evaluated with one-way ANOVA followed by Tukey’s post hoc test. A *p* < 0.05 was considered significant.

## Figures and Tables

**Table 1:** Experimental groups of Balb/c mice.

Name	*n*	Treatments
Control	20	No treatment
PTZ	20	PTZ administration
MPU	10	Treatment with *Mentha pulegium*
MPU + PTZ	10	Pre-treatment with *Mentha pulegium* prior to PTZ administration
MSW	10	Treatment with *Mentha spicata* wild
MSW + PTZ	10	Pre-treatment with *Mentha spicata* wild prior to PTZ administration
LAN	10	Treatment with *Lavandula angustifolia*
LAN + PTZ	10	Pre-treatment with *Lavandula angustifolia* prior to PTZ administration
ODI	10	Treatment with *Origanum dictamnus*
ODI + PTZ	10	Pre-treatment with *Origanum dictamnus* prior to PTZ administration
MPI	10	Treatment with *Mentha piperita*
MPI + PTZ	10	Pre-treatment with *Mentha piperita* prior to PTZ administration

## Data Availability

The original contributions presented in the study are included in the article/Supplementary Materials; further inquiries can be directed to the corresponding author/s.

## Supplementary Materials

The following supporting information can be downloaded at: https://www.mdpi.com/article/10.3390/ijms26199618/s1.

## Abbreviations

The following abbreviations are used in this manuscript:

EOs	Essential Oils
PTZ	Pentylenetetrazol
ROS	Reactive Oxygen Species
DSC	Differential Scanning Calorimetry
MDA	Malondialdehyde
LPO	Lipid Peroxidation
AOP	Antioxidant Power
DPPH	2,2-Diphenyl-1-picrylhydrazyl
FFPEs	Formalin-Fixed, Paraffin-Embedded Tissues
