# Supplementary figures and images for: Protective Effect of Aromatic Plant Essential Oil Administration on Brain Tissue of PTZ-Treated and Non-Treated Mice
Source: Int J Mol Sci. 2025 Oct 2;26(19):9618. doi: 10.3390/ijms26199618 (PMC12524642; doi:10.3390/ijms26199618)

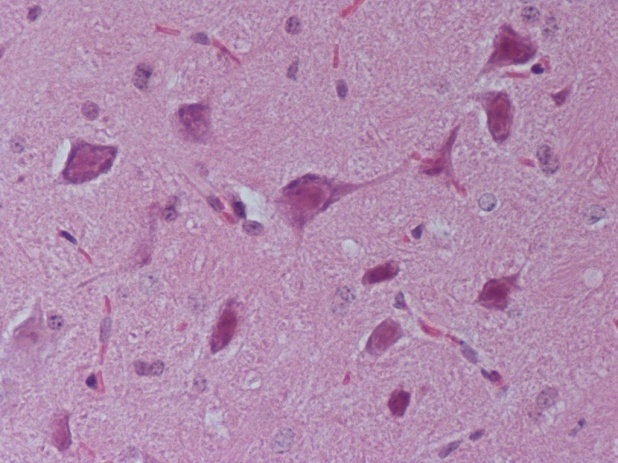

Supplement: Supplementary file 1 [file ijms-26-09618-s001.zip › a_Control.tif]

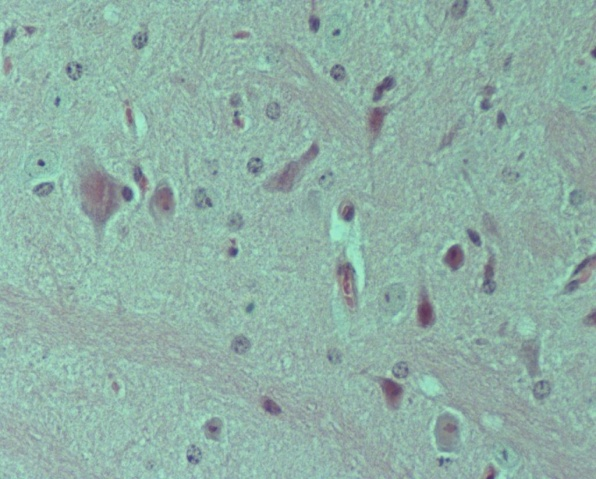

Supplement: Supplementary file 1 [file ijms-26-09618-s001.zip › b_PTZ group.tif]

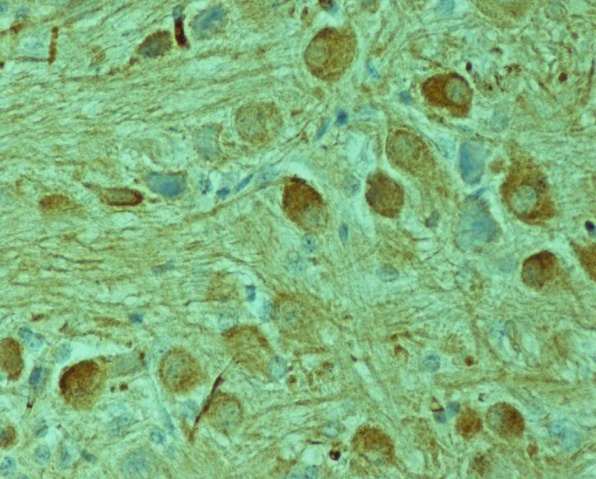

Supplement: Supplementary file 1 [file ijms-26-09618-s001.zip › c_Mentha piperita.tif]

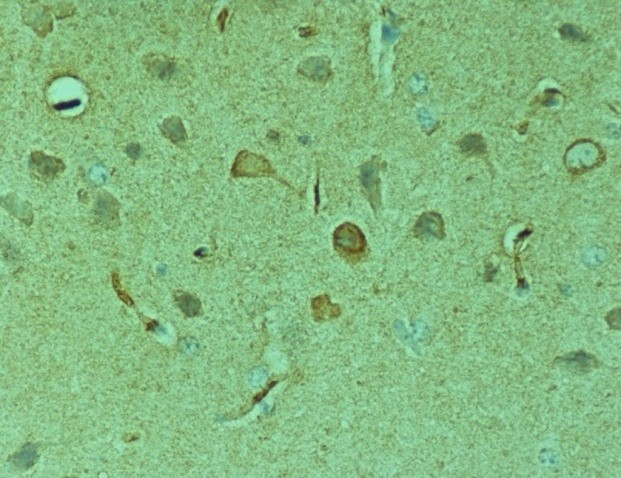

Supplement: Supplementary file 1 [file ijms-26-09618-s001.zip › d_Mentha Spicata wild.tif]

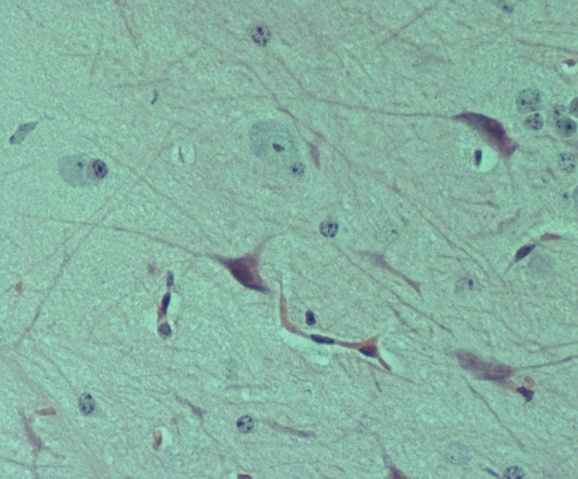

Supplement: Supplementary file 1 [file ijms-26-09618-s001.zip › e_Origanum dictamnus.tif]

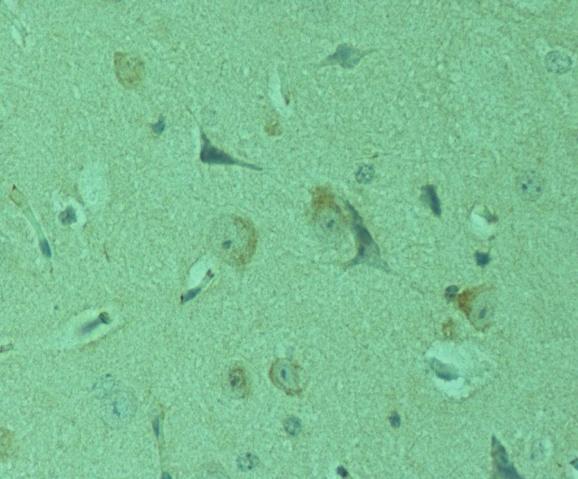

Supplement: Supplementary file 1 [file ijms-26-09618-s001.zip › f_Mentha pulegium.tif]

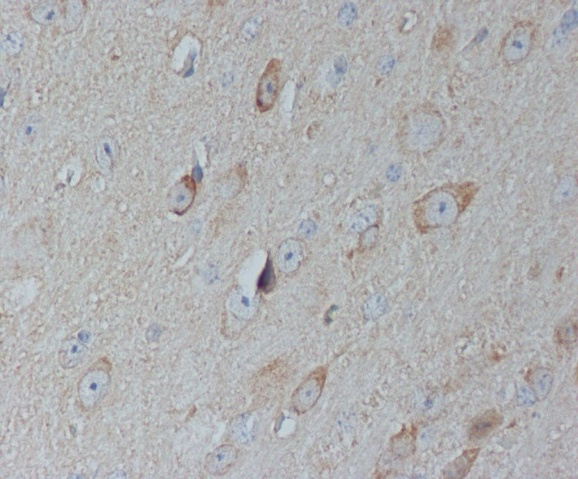

Supplement: Supplementary file 1 [file ijms-26-09618-s001.zip › g_Lavandula angustifolia.tif]
